# A clearer view of Southern Ocean air–sea interaction using surface heat flux asymmetry

**DOI:** 10.1098/rsta.2022.0067

**Published:** 2023-06-26

**Authors:** Simon A. Josey, Jeremy P. Grist, Jennifer V. Mecking, Ben I. Moat, Eric Schulz

**Affiliations:** ^1^ National Oceanography Centre, Southampton SO14 3ZH, UK; ^2^ Bureau of Meteorology, Melbourne, Victoria, Australia

**Keywords:** air–sea heat flux, wind stress, Southern Ocean

## Abstract

Progress in understanding Southern Ocean heat exchange and wind forcing is discussed and new results presented. These include a metric of the zonal asymmetry between surface ocean heat gain in the Atlantic/Indian sector and heat loss in the Pacific sector. The asymmetry arises from an intersector variation in the humidity gradient between the sea surface and near-surface atmosphere. This gradient increases by 60% in the Pacific sector enabling a 20 Wm^−2^ stronger latent heat loss compared with the Atlantic/Indian sector. The new metric is used for intercomparison of atmospheric reanalyses and CMIP6 climate simulations. CMIP6 has weaker Atlantic/Indian sector heat gain compared with the reanalyses primarily due to Indian Ocean sector differences. The potential for surface flux buoys to provide an observation-based counterpart to the asymmetry metric is explored. Over the past decade, flux buoys have been deployed at two sites (south of Tasmania and upstream of Drake Passage). The data record provided by these moorings is assessed and an argument developed for a third buoy to sample the Atlantic/Indian sector of the asymmetry metric. To close, we assess evidence that the main westerly wind belt has strengthened and moved southward in recent decades using the ERA5 reanalysis.

This article is part of a discussion meeting issue 'Heat and carbon uptake in the Southern Ocean: the state of the art and future priorities'.

## 1. Introduction

**Figure 1.**
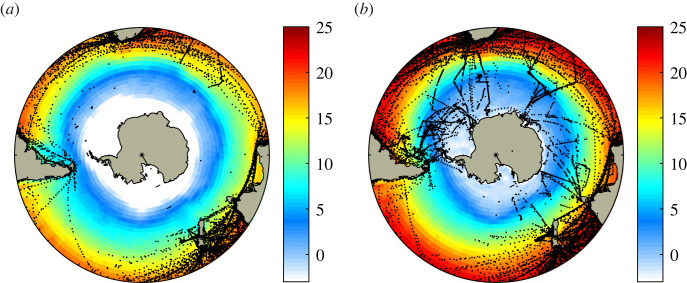
Distribution of ship observations in the International Comprehensive Ocean-Atmosphere Data Set [1] with sufficient variables to estimate the latent heat flux over the 5-year period 2000–2004 for (*a*) July, (*b*) January. Each point represents one latent heat flux estimate, note the extreme sparsity of observations in the Southern Ocean. Underlying colour field is NCEP/NCAR reanalysis SST, white indicates sea ice. (Online version in colour.)

The Southern Ocean has remained largely uncharted water for many decades because of its remoteness and the hostile conditions encountered. This is particularly the case for ocean–atmosphere interaction where even the sign of the long-term mean air–sea heat exchange is still uncertain in some regions. The frequency of observations required to estimate the different components of the heat exchange is exceptionally low in winter and barely adequate in summer (figure 1). Attempts have been made to determine the climatological surface heat flux field as part of global scale analyses but these have shown a high degree of dispersion (e.g. [2]). However, the situation is changing for the better with the advent of new observational capability, including long-term air–sea flux buoy reference site deployments [3–5], and potentially more reliable atmospheric reanalysis datasets. In addition to reanalyses, satellite observation-based datasets also offer relatively complete spatial coverage of the Southern Ocean in recent decades but are limited by the difficulty in accurately retrieving key near-surface meteorological fields needed to determine the heat flux (see §2).

It has been recognized for some time that, in the annual mean, much of the Atlantic/Indian sector of the Southern Ocean gains heat from the atmosphere while the Pacific sector loses heat [6–8]. In particular, [8] (T16, hereafter) explore this heat flux asymmetry in the context of the Antarctic Circumpolar Current (ACC) heat budget using the 1/6° Southern Ocean State Estimate (SOSE, [9]). In a simplified picture, T16 find that the surface heat gain in the Atlantic/Indian sector of the ACC is mostly balanced by cooling due to Ekman heat transport divergence. By contrast, in the ACC Pacific sector, cooling at the ocean surface reinforces that due to divergence of the Ekman heat transport and the warming required to balance the heat budget arises from convergent geostrophic heat advection.

Here, we examine progress made over the past decade and present new results which enable a clearer view of Southern Ocean air–sea interaction to be obtained. In particular, we build on the analysis of T16 with our focus being the potential of a zonally defined heat flux asymmetry metric for evaluation of atmospheric-reanalysis based surface flux datasets and climate models. We also consider the dependence of surface heat exchange on ocean model resolution in the context of projections of future warming and possible strengthening of Southern Ocean zonal flow due to an enhanced meridional SST gradient. Turning from models to observations, advances made with surface flux buoy reference site deployments over the past decade will be considered and the potential for an extended mooring network to provide an observation-based counterpart to the asymmetry metric explored. In the final section, we shift focus to wind forcing of the Southern Ocean and examine whether the main westerly wind belt has strengthened and—more controversially—moved southwards over the past 70 years.

## 2. Datasets and methodology

### (a) Datasets

We employ monthly mean surface heat exchange, near surface meteorology and sea surface temperature fields from the recent European Centre for Medium Range Weather Forecasting (ECMWF) Reanalysis 5 (ERA5, [10]) for the core of our analysis. ERA5 is at the high resolution (0.25° × 0.25°) end of global reanalyses currently available and has a pattern of Southern Ocean surface heat exchange that is consistent (see §3a) with results from SOSE (the leading ocean reanalysis for this region). In addition to ERA5, we determine the asymmetry metric for a range of other atmospheric reanalyses that include: the twentieth Century Reanalysis version 3 (20CRV3, [11]), ERA-Interim [12], Japanese 25-year Reanalysis (JRA-25, [13]) and National Centers for Environmental Prediction/National Centers for Atmospheric Research (NCEP/NCAR, also known as NCEP1, [14]). Note that all of the reanalysis models considered in our analysis assimilate satellite data with the exception of 20CRv3, which was designed to just assimilate *in situ* atmospheric pressure data. A common reference period of 1981–2010 is employed to determine the means required for the metric calculation (with the exception of JRA-25, for which we use the slightly shorter period 1981–2007). We also calculate the metric for historical simulations within the Coupled Model Intercomparison Project Phase 6 (CMIP6, [15]) again using the 1981–2010 period. To compute the CMIP6 multimodel mean heat flux field, a weighted average was employed that takes into account the number of ensemble members for each model, i.e. if there is only one ensemble member for a model that member is given a weight of 1, but if there are *n* ensemble members for a model then each member is given a weight of 1/*n*.

### (b) Southern Ocean sampling problem

From the perspective of air–sea interaction, the Southern Ocean is a data desert. In order to estimate the different components of the net air–sea heat exchange from ship observations, measurements are required of SST and near surface atmosphere conditions (air temperature, humidity and wind speed) for the turbulent heat flux components as well as cloud cover for the radiative fluxes. Historically, these have been provided by merchant ship observations, which reach a reasonable density in regions such as the North Atlantic and North Pacific that are spanned by major shipping routes (e.g. [16]). However, such information is rare to non-existent over much of the Southern Ocean, as illustrated in figure 1. The figure shows individual observations within the International Comprehensive Ocean-Atmosphere Data Set [1,17,18] that have the variables needed to estimate the latent heat flux over an example 5-year period (2000–2004). After this period, the number of Voluntary Ship Observations has tended to decline so the present day sampling is likely even lower than that shown. The frequency of observations is exceptionally low in winter and even in summer there are large regions with no observations, e.g. much of the Pacific sector. The impact of low sampling on air–sea fluxes is determined by two factors, namely the number of observations available for computing the flux and the magnitude of the local synoptic and mesoscale variability [19]. In the Southern Ocean, both factors contribute to the sampling problem as the variability terms are large, in contrast to tropical and subtropical regions where synoptic variability is smaller.

This situation remains the case today, despite the advent of satellite observations of many key ocean fields since the 1980s, as remote sensing from space cannot yet provide observations of the near surface air temperature and humidity variables at sufficient accuracy to reliably determine the turbulent (latent and sensible) surface heat exchange. This observation deficit makes the limited deployments of air–sea flux reference site buoys that have been possible in the Southern Ocean an extremely valuable resource. By providing measurements of air temperature and humidity they enable the sea–air temperature and humidity gradients to be quantified and thus *in situ* observation-based estimates of the heat flux throughout the annual cycle to be obtained for the first time. Two flux buoys have been deployed to date, one to the south of Tasmania and the other to the west of Drake Passage, and will be discussed in detail in §3b. It is important to note that progress toward better understanding of Southern Ocean air–sea interaction will benefit from synthesis of data from many sources. Thus, although we have stressed the value of air–sea flux moorings for making measurements of fields (air temperature and humidity) that are difficult to measure from space, we see a strong role for satellite measurements of SST and wind speed in synthesis studies.

### (c) Heat flux components

For the asymmetry analysis, we decompose the net heat flux (*Q*_n_) into its turbulent (*Q*_tur_) and radiative (*Q*_rad_) components.

2.1 $$Q_{\text{n}}=Q_{\text{tur}}+Q_{\text{rad}}.$$

Here the turbulent flux is simply the sum of the latent (*Q*_e_) and sensible (*Q*_h_) heat flux and the radiative flux is the sum of shortwave (*Q*_sw_) and longwave (*Q*_lw_) components. Our sign convention is for ocean heat gain/loss to be positive/negative. The primary contributors to the heat flux asymmetry (see §3a) are *Q*_e_ and *Q*_h_ so we focus on those terms rather than the radiative fluxes. *Q*_e_ and *Q*_h_ depend on near surface gradients of humidity (for latent) and temperature (for sensible) together with the wind speed through the following formulae:

2.2 $$Q_{\text{e}}=\rho LC_{\text{e}}\text{u}(q_{\text{s}}-q_{\text{a}})$$

and

2.3 $$Q_{\text{h}}=\rho {\text{c}}_{\text{p}}C_{\text{h}}\text{u}(T_{\text{s}}-(T_{\text{a}}+\gamma z)).$$

Here, *ρ* is air density; *L*, latent heat of vaporization; *C*_e_ and *C*_h_, latent and sensible heat transfer coefficients; *u*, scalar wind speed; *q*_s_, 98% of saturation specific humidity at sea surface temperature; *q*_a_, near surface atmospheric specific humidity; *c*_p_, specific heat capacity of air at constant pressure; *T*_s_, sea surface temperature (SST); *T*_a_, near surface air temperature with a correction for the adiabatic lapse rate, *γ*; and *z*, the measurement height for air temperature. Near surface variables presented in the text are at a height of 2 m for temperature and humidity and 10 m for wind speed. Finally, note that for the analysis presented in this paper we use fluxes provided in the reanalysis or CMIP6 model output rather than recomputing with equations (2.2)–(2.3), which are shown above with the purpose of drawing attention to the key driving terms.

## 3. Results

### (a) Asymmetry in the Southern Ocean heat exchange

#### (i) Large-scale structure

**Figure 2.**
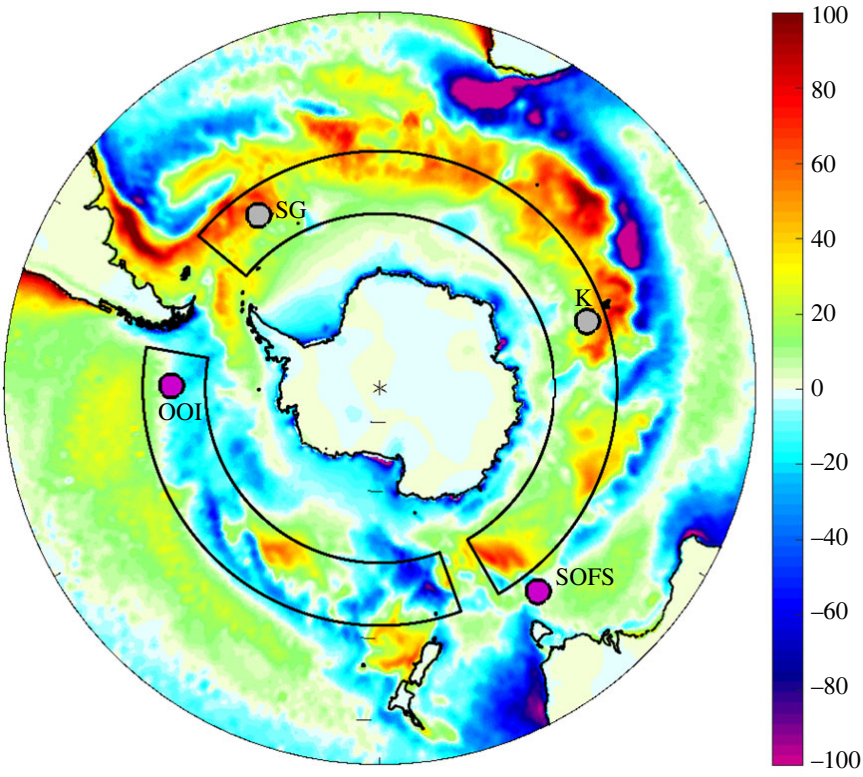
ERA5 1981–2010 annual mean net air–sea heat flux, *Q*_n_, units Wm^−2^. Grey boxes outline the regions used to define the heat flux asymmetry metric. The locations of the SOFS and OOI surface flux buoy deployments are shown by purple circles; possible future sites close to Kerguelen (K) or South Georgia (SG) are indicated by grey circles. (Online version in colour.)

The Southern Ocean has a strong seasonal cycle in *Q*_n_ driven largely by dominance of *Q*_sw_ in summer and *Q*_e_ in winter. These seasonal shifts cancel to a large degree leaving an annual mean net heat exchange field (figure 2) with a high degree of small-scale spatial structure that varies depending on the dataset under consideration. The main large-scale feature is the contrast between predominantly net ocean heat gain (strongest values for *Q*_n_ approaching 80 Wm^−2^) in the Atlantic-Indian sector of the Southern Ocean and a weaker region of primarily heat loss in the Pacific sector. We illustrate this feature for ERA5 but it is also evident in other studies with different datasets including the coarser (approx. 1.5°) resolution NCEP/NCAR reanalysis [6] and the Southern Ocean State Estimate (1/6°,T16) which has comparable resolution to ERA5 (1/4°).

T16 explore the asymmetry in the context of the ACC heat budget and consider the difference between Atlantic/Indian and Pacific ACC sectors defined according to contours of sea surface height. These sectors differ in the latitude range occupied by the ACC as it takes a more southerly path in the Pacific sector. Here, our focus is variations between flux datasets over a constant latitude band, rather than the ACC. So, we employ a different metric which measures the degree of asymmetry within a fixed latitude range. An advantage of adopting a fixed range is that the metric primarily reflects differences in the turbulent fluxes and largely avoids the influence of variations in the shortwave flux. The shortwave has a strong meridional variation that can play a significant role if the sectors have different latitude ranges as noted by Song [20] who carried out a streamline-based assessment of the surface heat flux variation over the ACC. The intention with the new metric is to probe the zonal asymmetry in the turbulent heat flux terms and thus potentially evaluate the ability of coupled models and atmospheric reanalyses to represent this large-scale aspect of Southern Ocean air–sea interaction.

With the above points in mind, we define the following metric of heat flux asymmetry between fixed latitude Atlantic/Indian and Pacific sectors of the Southern Ocean,

3.1 $$\Delta Q_{\text{n}}=Q_{\text{nAI}}-\;Q_{\text{nP}}.$$

**Figure 3.**
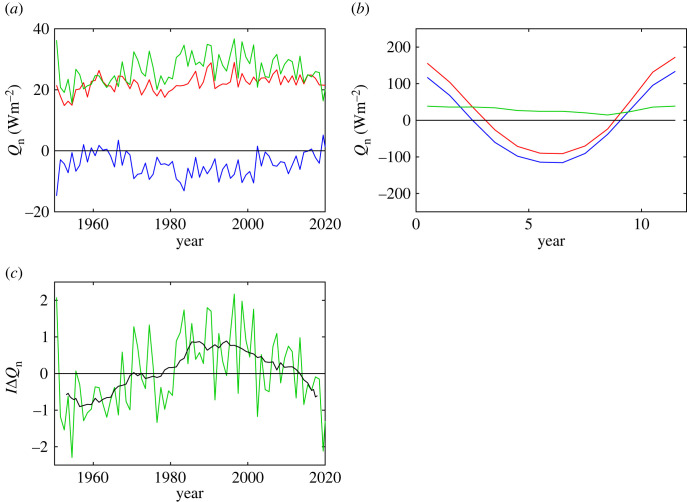
(*a*) Time series of ERA5 annual mean *Q*_n_ averaged over the Atlantic/Indian sector (*Q*_nAI_, red), Pacific sector (*Q*_nP_, blue) and their difference which is defined to be the asymmetry metric (Δ*Q*_n_, green). (*b*) Climatological monthly mean values for *Q*_nAI_, *Q*_nP_ and Δ*Q*_n_. (*c*) Time series of the ERA5 zonal heat flux asymmetry index, $I\Delta Q_{\text{n}}$ (annual mean values, green; 10-year running mean, black). (Online version in colour.)

Here, the subscripts AI and P refer to the area weighted mean of Q_n_ taken over the grey boxes shown in figure 2 which sample the main regions of heat gain and loss. The Atlantic/Indian sector box is (50°S–60°S, 50°W–150°E) and the Pacific sector box is (50°S–60°S, 160°E–80°W). Likewise, we have calculated corresponding asymmetry metric values for the heat flux components, the SST and near surface meteorological variables. Time series of *Q*_nAI_, *Q*_nP_ and Δ*Q*_n_ are shown in figure 3 for ERA5, they indicate that the Δ*Q*_n_ metric is robust over time lying typically in the range 25–35 Wm^−2^. The 1981–2010 climatological mean values are *Q*_nAI_ = 23.3 ± 2.4 Wm^−2^, *Q*_nP_ = −6.2 ± 3.4 Wm^−2^ and Δ*Q*_n_ = 29.5 ± 4.1 Wm^−2^ (where the ± values are the standard deviation of the individual annual means over the same period). We have also explored whether Δ*Q*_n_ has a seasonal dependence by determining climatological monthly mean values. The contributions from the Atlantic/Indian and Pacific sectors have similar large amplitudes of order ±100 Wm^−2^. However, their difference is relatively constant throughout the year with minor variations arising from a slight phase difference in *Q*_nAI_ and *Q*_nP_.

In addition to the Δ*Q*_n_ metric, which we employ subsequently for comparing different reanalyses and models (§3a(ii)), an index of year-to-year variations in the asymmetry strength for a given dataset can be defined as:

3.2 $$I\Delta Q_{\text{n}}=(\Delta Q_{\text{n}}-\;<\Delta Q_{\text{n}}>)/\sigma (\Delta Q_{\text{n}}),$$

where <> indicates the mean over all Δ*Q*_n_ values within a given period and *σ*(Δ*Q*_n_) their standard deviation. Individual yearly values for IΔ*Q*_n_ have been calculated for ERA5 (with mean and standard deviation determined using the 70-year period 1951–2020) and are shown in figure 3*c* together with their 10-year running mean. The figure shows notable variability at multi-decadal timescales with a shift from predominantly negative values (i.e. weaker asymmetry) pre-1980, positive values (stronger asymmetry) from approximately 1980–2010 and weaker values in recent years. The implications of this long-term variability remain to be explored but possible consequences include variations in the relative strengths of mode water formation between the Atlantic/Indian and Pacific sectors. We have also investigated whether the asymmetry index, IΔ*Q*_n_, has any dependence on the Southern Annular Mode (SAM) by correlating annual values with the Marshall [21] index (obtained from https://legacy.bas.ac.uk/met/gjma/sam.html) for the period of mutual overlap (1957–2020). The correlation is weak, *r* = 0.25, indicating that the asymmetry index potentially provides a useful measure of zonal Southern Ocean heat flux variability between the Atlantic/Indian and Pacific sectors in addition to the well-known meridional variations in the pressure field captured by the SAM index [21].

**Figure 4.**
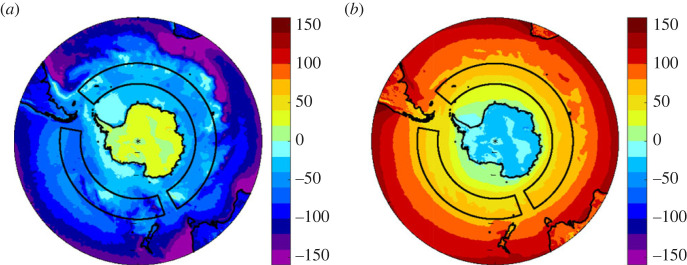
ERA5 climatological annual mean (*a*) turbulent and (*b*) radiative heat flux, units Wm^−2^. Grey boxes outline the regions used to define the heat flux asymmetry metric. (Online version in colour.)

Decomposition of the contributions to Δ*Q*_n_ from the different flux components reveals that it is primarily driven by the latent heat flux (Δ*Q*_e_ = 20.7 ± 2.3 Wm^−2^) with smaller contributions (3–5 Wm^−2^) from the sensible and longwave flux terms and no significant contribution from the shortwave (table 1). This is consistent with the turbulent and radiative heat flux fields shown in figure 4 which show stronger turbulent losses in the Pacific sector compared with the Atlantic/Indian sector while the radiative flux is similar in both. Annual mean values for the sea surface and meteorological variables in each sector and their difference are listed in table 2. These reveal that the primary driver for the stronger latent heat loss is the sea–air humidity difference (*q*_s _− *q*_a_), which increases by 63% from 0.8 g kg^−1^ in the Atlantic/Indian to 1.3 g kg^−1^ in the Pacific sectors. Note the corresponding increase in magnitude of the latent heat loss from 33.5 to 54.2 Wm^−2^ is 61%. The increase in humidity gradient reflects the warmer SST and air temperature in the Pacific sector and the greater moisture holding capacity at warmer temperatures arising from the nonlinearity of the Clausius-Clapeyron equation. It is also necessary to consider the wind speed as this may also play a role if the winds are stronger in the Pacific sector. However, the wind speed is very similar in the Pacific (10.6 ± 0.4 m s^−1^) and Atlantic/Indian (10.5 ± 0.2 m s^−1^) sectors. Thus, the flux asymmetry is a feature of the changing humidity difference, in turn influenced by the rising SST, rather than any change in the winds.

**Table 1.** Climatological (1981–2010) annual mean values of the heat flux components and the net heat flux for the Atlantic/Indian (AI) and Pacific (P) sectors together with their difference (AI-P).

variable	AI	P	Δ*Q*_x_ (AI-P)
*Q*_e_ (Wm^−2^)	−33.5 ± 1.2	−54.2 ± 2.2	20.7 ± 2.3
*Q*_h_ (Wm^−2^)	−6.4 ± 1.0	−11.5 ± 0.8	5.2 ± 1.2
*Q*_sw_ (Wm^−2^)	106.5 ± 1.4	106.1 ± 0.9	0.4 ± 1.6
*Q*_lw_ (Wm^−2^)	−43.3 ± 1.0	−46.6 ± 1.3	3.3 ± 1.5
*Q*_n_ (Wm^−2^)	23.3 ± 2.4	−6.2 ± 3.4	29.5 ± 4.1

**Table 2.** Climatological (1981–2010) annual mean values of sea surface temperature and near surface meteorological variables for the Atlantic/Indian (AI) and Pacific (P) sectors together with their difference (AI-P).

variable	AI	P	AI-P
*T*_s_ (°C)	1.8 ± 0.1	6.0 ± 0.3	−4.2 ± 0.2
*T*_a_ (°C)	1.1 ± 0.1	5.2 ± 0.2	−4.1 ± 0.3
*T*_s_ − *T*_a_ (°C)	0.7 ± 0.1	0.8 ± 0.1	−0.1 ± 0.1
*q*_s_ (g kg^−1^)	4.3 ± 0.0	5.8 ± 0.1	−1.5 ± 0.1
*q*_a_ (g kg^−1^)	3.5 ± 0.0	4.5 ± 0.1	−1.0 ± 0.1
*q*_s_ − *q*_a_ (g kg^−1^)	0.8 ± 0.0	1.3 ± 0.1	−0.5 ± 0.1
*u* (m s^−1^)	10.5 ± 0.2	10.6 ± 0.4	−0.1 ± 0.3

#### (ii) Variation across atmospheric reanalyses and coupled models

**Figure 5.**
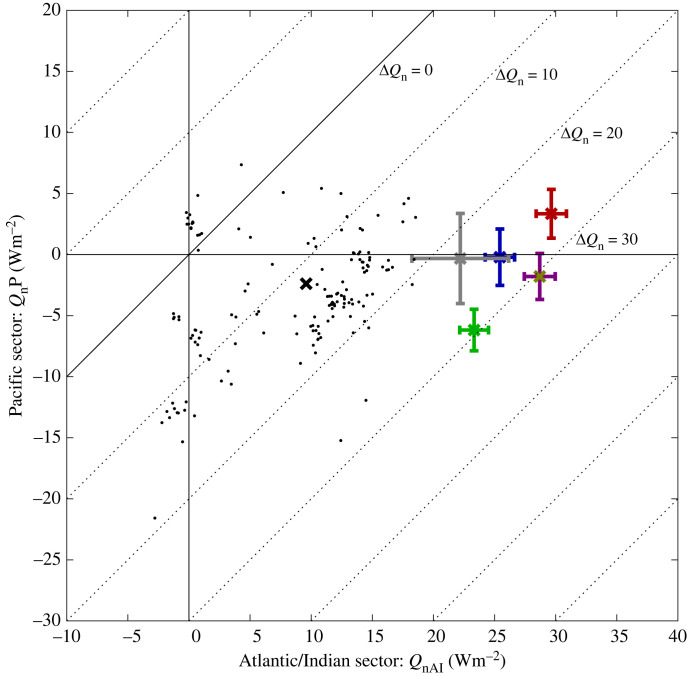
Scatter plot of Pacific sector net heat flux (*Q*_nP_) against Atlantic/Indian sector net heat flux (*Q*_nAI_) with the level of asymmetry (Δ*Q*_n_) indicated by dashed lines for various reanalyses (ERA5, green; ERAI, blue; JRA, purple; 20CRv3, red; NCEP/NCAR, grey) and the set of CMIP6 historical simulations (individual simulations, black points; multi-model mean, black cross). The one standard deviation error bars on the reanalysis estimates show the degree of inter-annual variability within the common 1981–2010 analysis period. For model details see the electronic supplementary material, table S1 and Eyring *et al.* [15]. (Online version in colour.)

The relatively small inter-annual variability of Δ*Q*_n_ relative to its mean value (figure 3) makes it a potentially useful measure of the climatological state of Southern Ocean air–sea interaction, and its representation in coupled models, with the potential to discriminate between the models. To explore this further, we show values for the metric and its components determined from various atmospheric reanalyses and CMIP6 historical simulations in figure 5. The solid diagonal line on this figure shows the case where the area averaged air–sea heat fluxes for the Pacific and Indian-Atlantic sector take the same value (the degree of asymmetry increases with distance away from this line). Each historical simulation is represented by a point on the figure; details enabling the particular simulation to be identified are provided in the electronic supplementary material, table S1. The atmospheric reanalysis datasets exhibit values for the asymmetry metric, Δ*Q*_n_, typically in the range 25–30 Wm^−2^. Within this narrow asymmetry range, differences in the heat flux in each sector are still observed. In particular, ERA5 and JRA display heat gain in the Atlantic-Indian sector and heat loss in the Pacific sector. By contrast, 20CRv3 has heat gain rather than loss in the Pacific sector and NCEP/NCAR shows stronger interannual variability than the other datasets considered. Note, our aim here is not to determine the causes of the variations between the reanalyses but rather to propose the asymmetry metric as a useful means of capturing large-scale differences in the Southern Ocean air–sea interaction regime between different datasets and models. We suggest it would form a useful addition to metrics already developed for other key variables [22].

**Figure 6.**
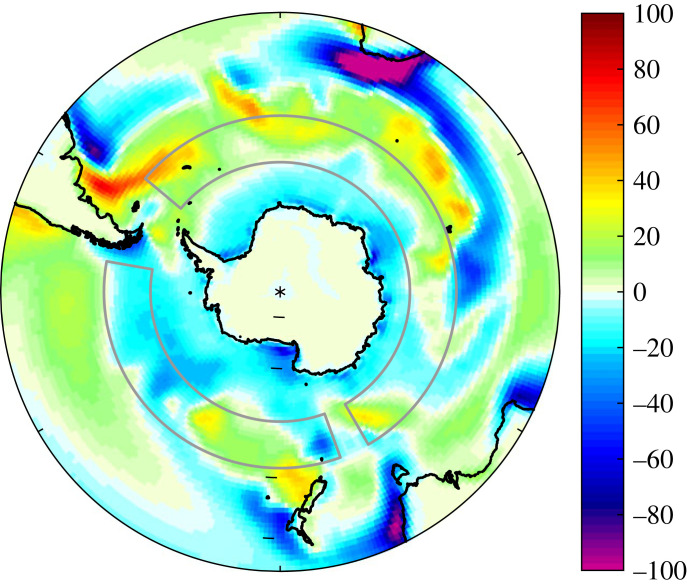
CMIP6 multi-model average annual mean net air–sea heat flux, *Q*_n_, units Wm^−2^. Grey boxes outline the regions used to define the heat flux asymmetry metric. (Online version in colour.)

Turning to the CMIP6 historical simulations, the majority have heat gain in the Atlantic/Indian and heat loss in the Pacific sectors. However, all of the simulations are offset from the observation-based atmospheric reanalyses, with notably weaker heat gain (typically in the range 0–20 Wm^−2^) or slight heat loss in the Atlantic/Indian sector. A subset of 38 out of 151 (25%) exhibit heat gain in the Pacific sector and a further small subset has Atlantic/Indian sector heat loss (20 of 151, 13%). The multi-model mean (black cross) is 9.6 Wm^−2^ gain in the Atlantic/Indian and −2.4 Wm^−2^ loss in the Pacific sector. Note, in forming the multi-model mean, we first averaged over ensemble members to form a mean for each model and then formed the multi-model mean by averaging over the resulting values including the models for which there was only a single simulation rather than an ensemble. Comparison of the multi-model mean field (figure 6) with ERA5 (figure 2) indicates that the CMIP6 models tend to capture the heat loss in the Pacific and the heat gain in the Atlantic sector but not the heat gain in the Indian sector. We plan to investigate the reasons for the CMIP6 model heat flux differences relative to the reanalyses in detail in a subsequent study and note here that they have potentially significant implications for the transformation of upwelled surface waters as they move northwards and form key water masses.

**Figure 7.**
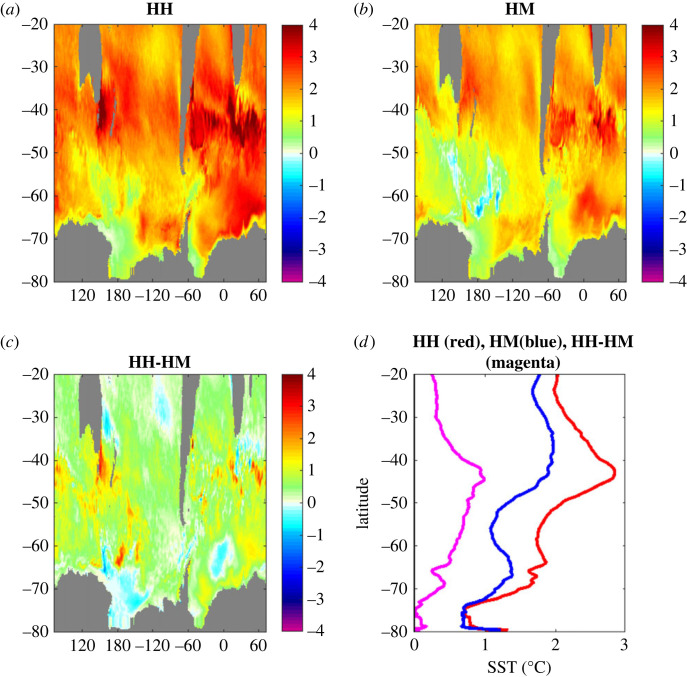
Projections of Southern Ocean SST change obtained with the HadGEM3-GC3.1 coupled climate model in 2031–2050 relative to 1950–1969 for (*a*) HH, (*b*) HM and (*c*) HH-HM. Panel (*d*) shows zonally averaged values of the fields presented in (*a–c*) with HH (red), HM (blue) and HH-HM (magenta). (Online version in colour.)

A further topic to be considered in future research is whether there is a dependence of coupled model air–sea heat exchange on model resolution. Resolution differences may influence both SST and near-surface atmospheric variables that control the heat flux (equations (2.2)–(2.3)) and is potentially of importance for climate projections. We illustrate this showing the SST increase (2031–2050 minus 1950–1969) for two configurations of the HadGEM3-GC3.1 coupled climate model which have a high (1/12°, labelled HH) and medium (1/4°, HM) resolution ocean component respectively, figure 7. The atmospheric model resolution is 25 km in each case. Note, the projections follow the SSP585 future scenario, for full details see Grist *et al*. [23]. The figure shows greater warming with the high-resolution ocean particularly at about 45°S, which is north of the Subantarctic Front. Thus, ocean resolution is potentially important for model-based studies of meridional SST gradient strength and consequently the strength of the zonal flow in the Southern Ocean. A combined model and observation-based analysis has found that this flow has recently accelerated due to a strengthening of the gradient [24]. Our results indicate that projections of whether this flow accelerates further in the decades ahead may be dependent on ocean model resolution. More generally, the relationship between the strength of heat flux asymmetry and model resolution, though beyond the scope of the current paper, merits further investigation in future work.

### (b) Surface flux buoy datasets

**Figure 8.**
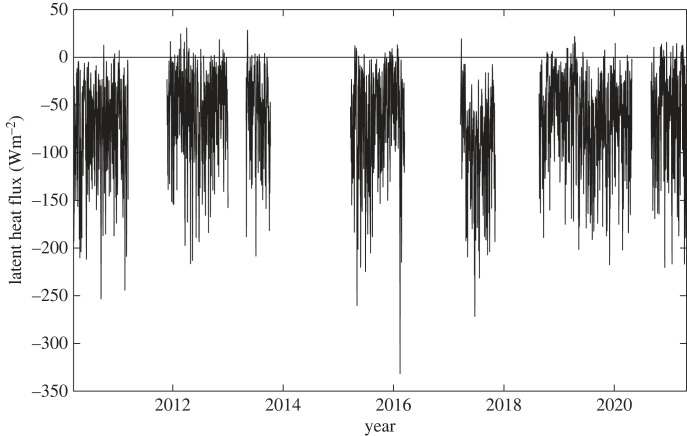
Daily mean latent heat flux heat flux, *Q*_e_, estimated from meteorological measurements made at the Southern Ocean Flux station mooring (units Wm^−2^) using the COARE Bulk Flux Algorithm version 3.5 (Fairall *et al*., 2003 [27]; Edson *et al*., 2013 [28]). Gaps show intervals where the mooring was not deployed.

The Southern Ocean is an extremely challenging environment for air–sea flux reference sites with a similar level of difficulty to that faced by deployments in other severe weather locations, e.g. the Irminger Sea [25]. The first successful air–sea flux buoy deployed in the region was the Southern Ocean Flux Station (SOFS) located to the south of Tasmania at 47°S, 142°E (figure 2 for site location) from March 2010 to March 2011 [4]. The wide range of variables observed by the buoy enabled the first accurate quantification of the annual cycle of net air–sea heat exchange and wind stress from a Southern Ocean location. The observed deployment mean net air–sea heat flux was a small net ocean heat loss of −10 Wm^−2^, varying from 139 Wm^−2^ (January) to −79 Wm^−2^ (July). SOFS also revealed a high degree of variability with daily mean turbulent heat loss as strong as −470 Wm^−2^, associated with cold southerly air flows. Subsequently, SOFS observations for March 2015–2016 were used in an assessment of two surface flux datasets [26]. Since the first deployment, SOFS has been deployed on a further 10 occasions including the current ongoing deployment. The overall data record is illustrated by the latent heat flux in figure 8 and spans 6 years within the 11-year period up to March 2021. This multi-year Southern Ocean surface flux record is unparalleled; it now provides the opportunity to determine valuable estimates of the climatological mean state of air–sea interaction at the SOFS site, as well as multi-annual variability, and to carry out long-term assessment of flux dataset accuracy. In particular, the time series could be used to evaluate atmospheric reanalyses (e.g. ERA5) at the SOFS location and we plan to undertake such an analysis in a separate study. Our aim in showing the time series here is to demonstrate the amount of data that is now available from SOFS and could be achieved at other sites in the Southern Ocean.

The second Southern Ocean flux buoy to be deployed was the Ocean Observatories Initiative (OOI) mooring sited west of Drake Passage at 54.5°S, 89.3°W from March 2015 to January 2020 with an occasionally interrupted data record within this period. The first 2.5 years of flux data from the deployment were analysed by Ogle *et al*. [3] followed by a study of simultaneous data from OOI and SOFS during periods of overlap [5]. A review of the findings from these deployments and their implications for mode water formation is provided by a companion paper in this special issue [29].

Consideration has also been given to where subsequent moorings could be deployed as part of a global network in Cronin *et al*. [30] and specifically within the Southern Ocean by Wei *et al*. [31]. The latter paper focused on capturing locations of strongest surface flux variability using the JRA reanalysis rather than trying to reduce uncertainty between flux datasets which is another important concern as demonstrated by the asymmetry metric. Wei *et al*. [31] put forward an ambitious plan for a ring of eight moorings (six in the Southern Ocean, two slightly outside to the north), which, if realized, would enable a major advance in our understanding of Southern Ocean air–sea interaction, particularly the drivers of within-region variability. In addition, there is a pressing need to resolve the reasons for the differences in the large-scale heat flux asymmetry between different datasets and coupled models that we have identified (§3a). These are of particular concern given the divergence between coupled model and atmospheric reanalysis estimates of heat gain in the Atlantic/Indian Ocean sector of the Southern Ocean (figure 5).

We propose that significant progress towards resolving these uncertainties could be achieved with multi-year observations from three surface flux buoy sites located at key points to sample the heat flux asymmetry. This approach would employ observations already made by SOFS and OOI and identify where additional deployments are needed. Note that the deployments do not have to be simultaneous and breaks in the record are acceptable; the key requirement is that each buoy is in the water for sufficiently long to generate robust climatological means for each month of the year. How many individual months are needed to reliably define a climatological month requires further work to establish but may be in the range 5–10 and thus nearly within reach at the SOFS site. More generally, it should be stressed that at whatever site is considered, a data record with gaps is still a valuable resource. In such a situation, 5–10 individual month observations within say a 15-year period potentially forms a useful estimate of the climatological monthly mean. However, non-stationarity of the surface meteorological and air–sea flux fields is an issue that needs to be considered if the record extends to multi-decadal timescales. Long-term time series without gaps still remains the goal but the reality of Southern Ocean mooring deployments to date is that there are gaps and thought needs to be given to how to make best use of such datasets. The value of long time series in other parts of the global ocean has been demonstrated in the recent study of Weller *et al*. [32], who evaluated different reanalyses and CMIP6 models against three surface flux reference moorings in Trade Wind forced regions. They found significant spatial variability in the differences between the moorings and the models with values of order 30 Wm^−2^ for both the reanalyses and CMIP6.

It is important to stress that the aim would be to use two of the three moorings to provide representative sites that enable a useful asymmetry metric to be determined as a two-point difference. We recognize that use of a two-site mooring-based metric will be limited but it will enable progress to be made toward resolving uncertainty over the strength of the asymmetry arising from the difference among and between the CMIP6 model and atmospheric reanalyses discussed earlier (figure 5). One of these two deployment sites should sit within the Atlantic/Indian Ocean sector and the other in the Pacific sector; the third site would then sample the transition region between the two. The existing SOFS buoy is well placed to sample this transition region and already spans a multi-year period. The Southern Ocean OOI site was well suited to represent the Pacific Ocean sector but is unfortunately no longer in the water and its existing record would need to be extended by redeployment to accurately characterize the annual mean regime at this location. The final site needs to be in the Atlantic/Indian Ocean sector to enable the annual mean heat flux in this regime to be estimated. To date, no flux buoys have been deployed in this broad region, hence we argue that a location in the Atlantic/Indian sector should form the highest priority for any future Southern Ocean flux reference site. Past experience suggests that potential deployment locations need to be relatively easily accessible from land as per the successful SOFS mooring. Hence, possible sites would include the Southern Ocean in the vicinity of Kerguelen, the area to the south of South Africa (although this is problematic because the atypical strong heat loss regime associated with the Agulhas current and its retroflection would need to be avoided) and the region close to South Georgia (probably to the east of this island to reduce potential iceberg hazard problems). Note that the new Atlantic/Indian mooring proposed here is required in addition to the existing SOFS mooring as SOFS lies in the transition zone between the Atlantic/Indian and Pacific sectors rather than being fully within the Atlantic/Indian sector. Research will also be needed to see how representative a given site is of the wider region in which it is located, potentially using transect measurements from other platforms (e.g. Saildrones) in dedicated campaigns centred on the mooring locations.

A further issue that the mooring data potentially enables to be addressed is the relationship between extreme fluxes and the mean field. Gulev & Belyaev [33] explored extreme fluxes in a reanalysis-based study and found that the strongest discrepancies between means and extremes occurred in the Southern Ocean. Further research is needed to determine the extent of these discrepancies and a combined reanalysis-surface flux buoy study could be expected to yield new insights in this area.

Many considerations arise in the development and execution of a successful and sustained observing effort beyond the science justification. Ideally, a proposed mooring site should be along or near an already established research vessel route/area of activity to minimize the logistical impact of such a long-term programme (as was the case with SOFS, which benefits from the Australian research vessel home port being close by). In addition, the mooring should be multi-disciplinary (rather than only concerned with physical fluxes) in order to maximize the benefit per unit cost and enable broad community uptake of the observations obtained. The mooring design and operation may dictate the ship capability needed for deployment and recovery; in this context, it is better to have a range of capable lower specification ships rather than being dependent on one highly in-demand vessel. Overdesigned moorings that can last for longer than 1 year without breaking are to be preferred in order to mitigate complications from lack of vessel availability or severe weather conditions preventing recovery/redeployment. However, for long deployments, consideration needs to be given to potential sensor failure, fouling, battery power and data storage, which are all issues that can limit success.

### (c) Changing Southern Ocean winds

**Figure 9.**
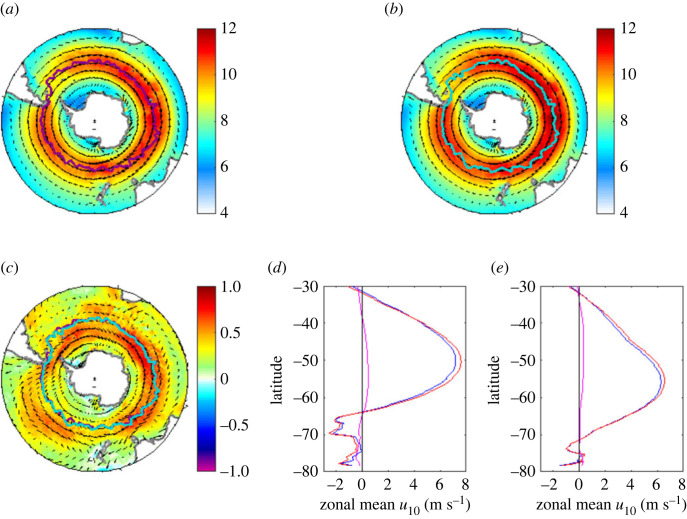
Climatological annual mean wind speed from ERA5 for (*a*) 1951–1985 and (*b*) 1986–2020. The location of the maximum zonal component of the wind speed at a given longitude is indicated by the thick solid lines (magenta in (*a*) and cyan in (*b*)). (*c*) The difference in wind speed between the two 35-year periods, 1986–2020 minus 1951–1985 (with the magenta and cyan lines from (*a*,*b*) reproduced to facilitate comparison). (*d,e*) The distribution of zonal wind speed as a function of latitude averaged over the (*d*) Atlantic/Indian sectors and (*e*) Pacific sector. In each case, blue shows the 1951–1985 distribution, red the 1986–2020 distribution and magenta their difference (1986–2020 minus 1951–1985). (Online version in colour.)

In this section, we shift from the heat exchange to consider the wind forcing of the Southern Ocean with a focus on multi-decadal changes in the winds. The dominant feature of the wind field in the Southern Ocean is the band of intense westerlies circling the region and peaking at 50–55°S (figure 9). These winds through the associated wind stress and equatorward Ekman transport play a key role in establishing the Southern Ocean circulation, in combination with the poleward transport due to eddies (note that winds play a further role via wave generation with the waves contributing via additional mixing). It has been well-established for some time that the winds have strengthened over the past 70 years through evaluation of atmospheric reanalysis data since the 1950s (e.g. [34,35]). Note that changes due to reanalysis assimilation of satellite data are probably not a major factor in the Southern Ocean wind strengthening trend as previous studies have shown the trend is also evident in the period (1950s–1980s) prior to the satellite era (e.g. [34]). Furthermore, there is medium confidence that boreal-winter storm tracks during the last decades experienced poleward shifts over the Southern Ocean [36]. However, it is also often stated that the main wind belt has moved southwards on similar timescales despite the evidence for such a shift being much weaker. Here, we assess the evidence for both strengthening and potential southward displacement of the westerlies and present an update using the ERA5 reanalysis.

A range of studies has built on the original analysis of Swart & Fyfe [34] and provided further evidence for strengthening of the wind belt although substantial meridional displacement of the belt is not clearly seen. Such studies have primarily employed zonal wind speed measures averaged over the full range of longitude. However, longitudinal variations have now been considered in two recent studies [37,38]. In particular, Waugh *et al*. [38] find using the ERA-Interim, JRA-55 and MERRA2 reanalyses that there are significant differences in behaviour between the Pacific and Atlantic/Indian regions of the Southern Ocean. In the Pacific, there is a large strengthening but only a weak, meridional shift which is equatorward rather than poleward. By contrast, in the Atlantic/Indian sector there is only a weak increase in wind strength but a significant poleward shift.

We have explored the extent of changes to the wind field using ERA5. Climatological annual mean wind speed fields from ERA5 for 1951–1985 and 1986–2020 are shown in figure 9 together with their difference (note [38] found significant seasonal variations but here for brevity we focus on the annual mean field). The band of strong westerlies is evident in both figures and the location of the maximum zonal component of the wind speed at a given longitude is indicated by the thick solid lines. These lines are reproduced in figure 9*c*, which shows the difference in wind speed between the two 35-year periods. There is a strengthening of the wind speed with stronger winds in the main belt and to its south (north) in the Atlantic/Indian (Pacific) sectors supporting the finding of differential change obtained by Waugh *et al*. [38]. There is little or no movement in the latitude of the wind speed maximum and this may reflect limitations in the use of peak wind speed as a measure of belt location. Instead, we suggest that the position of the belt is better represented by considering the distribution of zonal wind speed as a function of latitude averaged over the Atlantic/Indian and Pacific sectors (figure 9*d,e*). With this measure there is marginal evidence of a southward broadening of the belt in the Atlantic/Indian sector and northward broadening in the Pacific sector even though the latitude of maximum wind speed remains essentially unchanged. The main point to note from figure 9*d,e* is that the changes to the wind speed strength and latitude are small. This needs to be borne in mind, particularly with model studies, which in some cases have applied major perturbations to the wind speed to explore potential impacts on the Southern Ocean circulation ([37] note that earlier studies have imposed shifts of between 0.5 and 10 degrees of latitude and wind intensification factors of between 10% and 300%).

Why is there a difference in the wind speed changes between the Atlantic/Indian and Pacific sectors? Using climate model analysis, Waugh *et al*. [38] suggest that the differential behaviour is the result of internal variability rather than being a forced response to climate change. Given the Atlantic/Indian – Pacific heat flux asymmetry results that have been the main focus of our analysis, it is interesting to ask whether the differential wind speed behaviour found by Waugh *et al*. [38] is in some way linked to the different heat flux regimes experienced in the two sectors. It is not possible to answer this question at present but future coupled model experiments could potentially shed light on this issue.

## 4. Conclusion and discussion

In this paper, we have considered the large-scale properties of Southern Ocean air–sea heat exchange and wind forcing. The main features of the annually averaged heat exchange are heat gain in the Atlantic/Indian sector of the Southern Ocean and heat loss in the Pacific sector. The primary driver of this asymmetry is the intersector variation in the humidity gradient between the sea surface and near surface atmosphere. The humidity gradient increases by 60% in the Pacific sector enabling a 20 Wm^−2^ strengthening of the latent heat loss compared with the Atlantic/Indian sector; by contrast, wind speed variations are not a major factor.

We have developed a metric of the heat flux asymmetry using a fixed latitude range (50–60°S) and suggest that it can usefully be employed to characterize zonal variations in the large-scale air–sea heat exchange properties of the Southern Ocean. Specifically, we use the metric for an intercomparison of the surface heat flux in atmospheric reanalysis datasets and CMIP6 climate models. It reveals a clear separation between the datasets and the models, with the models tending to have a weaker level of asymmetry due to insufficient heat gain in the Atlantic/Indian sector. Further analysis is required to establish the reasons for the difference in asymmetry strength between the coupled models and the reanalyses; at this stage the primary issue appears to be variations in the heat exchange within the Indian Ocean sector. Understanding the drivers behind these differences has the potential to lead to more reliable surface flux datasets and thus enable advances in the study of Southern Ocean air–sea interaction and mixed layer properties; for example, determination of the causes of mixed layer heat content anomalies using an approach recently applied to the North Atlantic subpolar gyre [39,40].

The differences we have noted in the level of surface heat flux asymmetry in reanalyses and coupled models reveals the pressing need for high quality reference site observations in the Southern Ocean. Such observations are possible with air–sea flux moorings and two moorings, SOFS (south of Tasmania) and OOI (upstream of Drake Passage), have been deployed at various times in the past decade. SOFS has the longer data record, starting in 2010, and the data collected now represent a major resource for air–sea flux studies. OOI was deployed from 2015 through 2020, with interruptions, and also provides a valuable dataset, which if extended would enable a full determination of the climatological mean heat exchange in the Pacific sector. To date there has been no surface flux buoy in the Atlantic/Indian sector and a deployment within this region (potentially from South Georgia or Kerguelen) would be extremely valuable to accurately characterize the heat flux regime at a location within this pole of the asymmetry metric.

The wind forcing of the Southern Ocean has also been considered with an assessment of the evidence that the westerly wind belt has strengthened and moved southward in recent decades. The main development here has been the analysis by Waugh *et al*. [38], who find that longitudinal variations in the strength of any trends need to be considered. Their analysis reveals a large strengthening in the Pacific but only a weak, meridional shift which is equatorward rather than poleward. In the Atlantic/Indian sector there is only a weak increase in wind strength but a stronger poleward shift. We have extended their analysis using ERA5 and note that the use of peak wind speed values to identify meridional shifts in the wind belt should be supplemented by a consideration of the wind speed distribution with latitude. By considering this distribution it is evident that the winds in both sectors have experienced some strengthening but only marginal shifts in latitude. The drivers of these differences remain to be determined but it is clear that the wind field, like the heat flux, exhibits asymmetric properties in the Southern Ocean. Further analysis of these asymmetries and extension to other climatically important fields (particularly the freshwater and CO_2_ flux, which exhibits complex zonal variability) is likely to yield new insights into Southern Ocean air–sea interaction in the years ahead, at a time when improved understanding of this key component of the changing climate system is urgently required.

## Data Availability

This article includes data obtained from many different projects including a wide range of atmospheric reanalyses and CMIP6 climate simulations. Where articles are cited in reference to data, please see the referenced article and their data accessibility statements. The data are provided in the electronic supplementary material [42].
